# Machine Learning for Early Parkinson’s Disease Identification within SWEDD Group Using Clinical and DaTSCAN SPECT Imaging Features

**DOI:** 10.3390/jimaging8040097

**Published:** 2022-04-02

**Authors:** Hajer Khachnaoui, Nawres Khlifa, Rostom Mabrouk

**Affiliations:** 1 Laboratoire de Biophysique et Technologies Médicales, Institut Superieur des Technologies Medicales de Tunis, Université de Tunis El Manar, Tunis 1006, Tunisia; khalifa_nawres@yahoo.com; 2 Department of Computer Sciences, Bishop’s University, Bishop’s 2600 College St., Sherbrooke, QC J1M 1Z7, Canada; rostom.mabrouk@ubishops.ca

**Keywords:** Machine Learning, SPECT imaging, Parkinson’s Disease, SWEDD, clustering algorithms

## Abstract

Early Parkinson’s Disease (PD) diagnosis is a critical challenge in the treatment process. Meeting this challenge allows appropriate planning for patients. However, Scan Without Evidence of Dopaminergic Deficit (SWEDD) is a heterogeneous group of PD patients and Healthy Controls (HC) in clinical and imaging features. The application of diagnostic tools based on Machine Learning (ML) comes into play here as they are capable of distinguishing between HC subjects and PD patients within an SWEDD group. In the present study, three ML algorithms were used to separate PD patients from HC within an SWEDD group. Data of 548 subjects were firstly analyzed by Principal Component Analysis (PCA) and Linear Discriminant Analysis (LDA) techniques. Using the best reduction technique result, we built the following clustering models: Density-Based Spatial (DBSCAN), K-means and Hierarchical Clustering. According to our findings, LDA performs better than PCA; therefore, LDA was used as input for the clustering models. The different models’ performances were assessed by comparing the clustering algorithms outcomes with the ground truth after a follow-up. Hierarchical Clustering surpassed DBSCAN and K-means algorithms by 64%, 78.13% and 38.89% in terms of accuracy, sensitivity and specificity. The proposed method demonstrated the suitability of ML models to distinguish PD patients from HC subjects within an SWEDD group.

## 1. Introduction

PD is a progressive, irreversible and complicated brain disorder characterized by a combination of both motor and nonmotor symptoms, including tremor, rigidity, bradykinesia, postural instability, depression, sleep disturbances and olfactory issues [1,2]. One of the most common causes of this disease is the gradual neurodegeneration of dopaminergic neurons of the substantia nigra, which results in a diminution in the dopamine in the striatum and destruction of dopamine transporters (DaT) [3,4]. As the dopamine continues to decline, the disease progresses, while a significant change is taking place in the striatum’s shape [4]. By the time that PD signs become clinically detectable, the dopaminergic neurons are damaged. Indeed, PD progression starts before symptoms are clinically detected.

At present, there is no cure for PD and no way to restore neurons once they are destroyed, because the reason for the dopamine neurons’ death is still mysterious. However, with the help of certain drugs, the symptoms of this disorder can be controlled and the patient can continue his normal life with no further degradation of dopamine neurons [5]. At this stage, it is important to make an early and accurate diagnosis and identification of this disease to initiate neuroprotective therapies. However, early and accurate clinical diagnoses are complicated because they are only possible at a late stage when symptoms are obvious enough. Thus, the introduction of Single-Photon Emission Computed Tomography (SPECT) neuroimaging modality in PD diagnosis has improved the accuracy rate of predicting early PD disease. SPECT with 123I-Ioflupane (DaTSCAN) shows that there is a significant depletion of DaT in PD patients, even at an early stage. Indeed, 123I-Ioflupane is a popular radiotracer used for PD which has a high binding affinity for DAT. Consequently, DaTSCAN is a suitable diagnostic tool for early PD patients whose interpretation is visually carried out by experts [6]. Nevertheless, some patients are clinically diagnosed as having PD but have a normal imaging, a phenomenon termed as SWEDD [7,8]. After the follow-up period of SWEDD, some subjects of this group develop PD while others do not (Healthy Control (HC)) [7,8]. Consequently, early clinical PD, SWEDD and HC groups are mild and overlap. Thus, the separation of early PD patients and HC subject from the SWEDD group has become debatable, because early PD needs different strategies for therapeutic intervention. In recent years, a semi-quantification technique has been used in clinical practice to enhance visual reporting and interobserver variability by providing SBR values [9]. These values give neutral measures of dopaminergic function. However, the striatal uptake shape information and particular pattern are not reflected in the SBR results, which leads to a wrong early PD diagnosis. Hence, the semi-quantification technique, which is based only on imaging information, is a relatively limited tool for analyzing SPECT images. The limitations of this technique are resolved through the development of Computer-Aided Diagnosis and Detection (CADD) systems based on Machine Learning (ML) methods that receive several input features (clinical scores and SPECT imaging information). Thus, CADD systems have become popular with results surpassing standard benchmarks [10,11,12,13,14,15]. As in various medical applications, CADD systems are extensively used for PD diagnosis, often with effective findings [16,17,18,19]. Therefore, a number of ML algorithms were evaluated in order to find a useful and automated approach for early PD identification [17,18,19,20].

### 1.1. Related Works

In the literature, many studies applied ML algorithms to automate PD diagnosis. Most of them focused on using the SVM method and PPMI dataset in their research [21,22,23,24,25,26,27]. Indeed, these approaches outperformed conventional (visual interpretation and semi-quantification technique) data analysis tools. For instance, Diego et al. [21] performed an Ensemble Classification model that combines SVM with linear kernel to differentiate between PD patients and HC subjects. The dataset obtained from the PPMI consists of 388 subjects (194 HC subjects, 168 PD patients and 26 SWEDD subjects). PD and SWEDD subjects were both labeled as the PD group. Morphological features extracted from DaTSCAN images with biomedical tests were split into training and test sets. The proposed method’s performance was evaluated using the Leave-One-Out (LOO) Cross-Validation (CV) method and reached a high accuracy rate of 96%. Nicolas Nicastro et al. [24] applied the SVM method to identify PD patients from other parkinsonian syndromes and HC subjects using semi-quantitative 123-FP-CIT SPECT uptake values. Striatal Volumes-Of-Interest (VOIs) uptake, VOIs asymmetry indices and the caudate/putamen ratio were used as input for the proposed method. The latter was evaluated for 578 samples divided into 280 PD patients, 90 with other parkinsonian syndromes and 208 HC subjects (parkinsonian syndrome patients and HC subjects were considered to be in the same group) obtained from a local database. It achieved an accuracy rate of 58.4%, sensitivity of 45%, specificity of 69.9% and AUC of 60% using the five-fold CV technique. Additionally, Yang et al. [22] developed a two-layer stacking ensemble framework to classify PD patients and HC subjects. The data used in this study consisted of 101 subjects divided into 65 PD patients and 36 HC subjects, as obtained from PPMI dataset. The proposed method combined multimodel neuroimaging features composed of MRI and DTI with clinical evaluation. The formed multimodel feature set was the input for the first layer, which consists of SVM, Random Forests, K-nearest Neighbors and Artificial Neural Network. In the second layer, the Logistic Regression algorithm was trained based on the output of the first layer. The proposed method was evaluated using a ten-fold CV method and achieved an accuracy of 96.88%. Dotinga et al. [23] presented linear SVM to identify PD patients from non-PD patients. This approach was developed using eight striatal I-123 FP-CIT SPECT uptake ratios, age and gender as input features. These inputs of 210 subjects were split into three sets, which were training (90), validation (80) and testing (40). The proposed method performance was evaluated using the ten-fold CV technique and achieved an accuracy of 95%, a sensitivity of 69% and a specificity of 93.3%. Lavanya Madhuri Bollipo et al. [25] applied incremental SVM with the Modified Frank Wolfe algorithm (SVM-MEW) for early PD diagnosis and prediction using data from the PPMI dataset. The latter contained 600 samples, out of which 405 were early PD subjects and 195 were HC subjects divided into training and testing sets. Each sample was composed of 11 features of clinical scores, SBRs values and demographic information. The model’s optimal hyperparameters were obtained through 10-fold grid CV. The proposed method was used with several kernels: linear, polynomial, sigmoid, RBF and logistic functions. It was evaluated using the LOO-CV technique and reached an accuracy of 98.3%. Lavanya Madhuri Bollipo et al. [26] presented an optimized Support Vector Regression (SVR) algorithm to diagnose early PD and predict its progression. This algorithm was trained with weights associated with each of the sample datasets by giving 12 sets of features (motor, cognitive symptom scores and SBR). The dataset consisted of 634 subjects, out of which 421 were early PD and were 213 HC subjects, taken from the PPMI dataset. It was normalized for balancing the influence of each feature and divided into training and testing sets. The proposed method was used with linear, 4th order polynomial, sigmoid, Radial Basis Function (RBF) and logistic kernels. SVR with RBF kernel achieved the best accuracy of 96.73% in comparison with the other kernel functions. Diego Castillo-Barnes et al. [27] assessed the potential of morphological features computed from 123I-FP-CIT SPECT brain images to distinguish PD patients from HC subjects. A dataset of 386 samples obtained from the PPMI database and divided into 193 HC subjects and 193 PD subjects was used in this study. The optimal morphological features were selected using Mann–Whitney–Wilcoxon U-Test, and then classified through SVM, Naive Bayesian and Multilayer Perceptron (MLP) algorithms. The proposed method was evaluated using the ten-fold CV technique and achieved an accuracy of 97.04%.

These research works found that ML techniques have good potential for classification and help to improve the accuracy of PD diagnosis [21,22,23,24,25,26,27]. Classification methods, datasets and performance metrics of the related works are summarized in Table 1. However, the common issue in neuroimaging research is the high dimensionality of data. The feature reduction method is one of the most effective ways to solve this issue. It selects a relatively small number of the most representative, informative, relevant and discriminative subsets of features to construct reliable ML models. In addition, most of these studies focused on using the SVM method. Nevertheless, the latter is not suitable for large databases. It does not perform well when the classes in the database overlap. Moreover, in cases where the number of features for each data point exceeds the number of training data samples, the SVM underperforms.

### 1.2. Contributions

Motivated by the recent works that distinguish PD patients from HC subjects, and since the PD, SWEDD and HC groups overlap, we propose an unsupervised classification approach to differentiate between HC subjects and PD subjects within SWEDD groups, which is a more difficult task compared with previous studies. The proposed method is based on ML clustering models (DBSCAN, K-means and Hierarchical Clustering) and feature reduction methods (PCA and LDA). It will help medical practitioners in determining early PD diagnoses.

The key contributions and objectives of the proposed study are summarized as follows:

1. A diagnostic tool based on ML methods is proposed to improve the performance of early PD diagnosis within SWEDD groups, as the regular SWEDD subjects are likely to have PD at follow-up;
2. The PPMI dataset was used as an input (548 samples with nine features) for the proposed method, as it is a large database that includes healthy and unhealthy subjects from different locations, which adds diversity in the dataset and makes the proposed method robust. Those heterogeneous features are divided into four features derived from DaTSCAN SPECT images (SBR values of left and right caudate and putamen), and five scores were derived from computer clinical assessments (Unified Parkinson’s Disease Rating Scale (UPDRS III), Montreal Cognitive Assessment (MoCA), University of Pennsylvania Identification Test (UPSIT) State-Trait Anxiety Inventory (STAI) and Geriatric Depression Scale (GDS));
3. The optimum features were chosen from the nine features of the three groups (PD, HC and SWEDD) through PCA and LDA feature reduction algorithms to keep relevant information. This resulted in a reduction in the computational cost and improvement of the proposed method’s performance;
4. Clustering assessments were used to distinguish PD patients from HC subjects within SWEDD using DBSCAN, K-means and Hierarchical Clustering (the reduction technique result of the SWEDD group was used as input for these ML clustering algorithms);
5. The proposed model was evaluated for accuracy, specificity, sensitivity and F1 score by comparing clustering outcomes with the SWEDD ground truth (after follow-up, some SWEDD subjects developed PD, whereas other subjects continued to have normal dopaminergic imaging (HC)).

This research is organized as follows: Section 2 presents the proposed approach, which includes dataset information, feature reduction methods (Principal Component Analysis (PCA) and Linear Discriminant Analysis (LDA)) and clustering methods (Density-Based Spatial (DBSCAN), K-means and Hierarchical Clustering) as subsections. Section 3 describes the experimental results and findings. Section 4 provides the discussion, and Section 5 presents conclusions and future enhancements that can be elaborated upon beyond this work.

## 2. Materials and Methods

**Figure 1:**
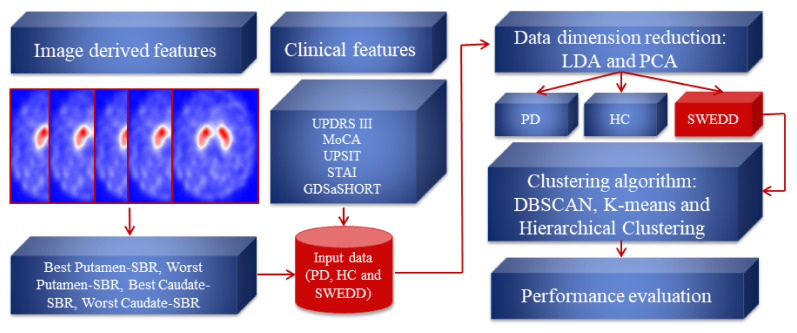
The structure diagram of the proposed method.

This section introduces the proposed method for identifying PD within an SWEDD group. As a first step, the dataset containing nine clinical and imaging features of 548 subjects was prepared. Then, the feature reduction techniques were performed to compute the projection matrices. The latter project the data to lower dimensions and generate 2D-LDA and 2D-PCA data vectors that identify primary symptoms. Following this, the feature reduction result with the best performance was used to build clustering models (DBSCAN, K-means and Hierarchical Clustering) to naturally self-organize the SWEDD samples into two groups (HC and PD). The evaluation was performed by comparing clustering outcomes with the ground truth (follow-up). Each step of the proposed method is explained in the subsequent subsection. Figure 1 presents the structure diagram of the proposed method. 

### 2.1. Dataset Description

Clinical and neuroimaging data sourced from the Parkinson’s Progression Markers Initiative (PPMI) database (http://www.ppmi-info.org/ (accessed on 4 January 2022)) were used in developing this study. PPMI is a partnership of scientists, investigators and researchers that are committed to assessing the evolution of clinical, imaging and biomarker data in PD patients. They are dedicated to building standardized protocols for acquisition and analysis of the data [28]. 

This research work explored 548 subjects divided into three classes (341 PD patients, 156 HC subjects and 51 SWEDD subjects) with clinical and DaTSCAN SPECT imaging data. SWEDD group was limited to 50 subjects who underwent 2-year follow-up scans. Each subject had nine features divided into five clinical features and four DaTSCAN SPECT imaging features. Information about each group is given in Table 2.

#### 2.1.1. SPECT Imaging Features: Striatal Binding Ratio (SBR)

**Figure 2:**
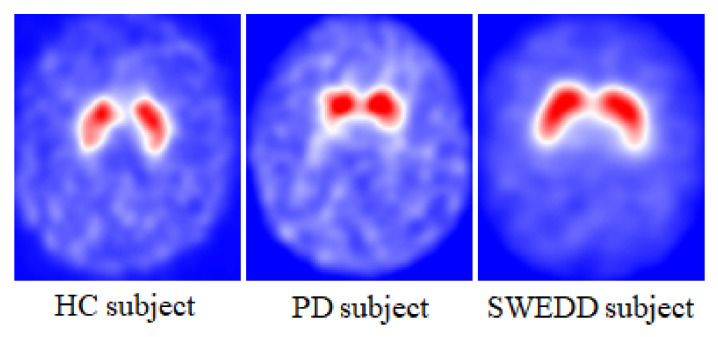
DaTSCAN SPECT imaging of the dopaminergic system for HC, PD and SWEDD subjects.

The SPECT neuroimaging data were acquired after radiopharmaceutical injection (123I-FP-CIT known as 123I-Ioflupane) with a target dose of 111–185 MBq. 123-Ioflupane is a ligand that binds the dopamine transporters in the striatum (putamen and caudate) structures [29,30]. PD patients are marked with smaller dopamine density in the striatum region, as shown in Figure 2. Prior to the injection, subjects were pretreated with a stable iodine solution to reduce the radiotracer uptake by the thyroid. Due to the various types of SPECT scanning equipment at different centers, PPMI uses a standardized scanning acquisition protocol. Image data were acquired in a 128 × 128 matrix. Then, these raw projections were iteratively reconstructed and the attenuation was corrected. The final preprocessed images were saved in DICOM format with dimensions of 91 × 109 × 91. These images were spatially and intensity-normalized according to the protocol of Montreal Neurologic Institute to ensure that any voxels in different images corresponded to the same anatomical position across the brain. These registrations were carried out using Statistical Parametric Mapping (SPM8) software for the spatial normalization and the Integral Normalization algorithm for the intensity normalization. Automated Anatomical Labeling (AAL) was used for the extraction of regional count densities in the left and right putamen and caudate. The SBR values of these four regions were calculated for each image with reference to the occipital cortex [28]. Data were organized as following: the best putamen and caudate had the highest SBR values, and the worst putamen and caudate had the lowest SBR values.

#### 2.1.2. Clinical Features

Several clinical measurements were used to evaluate PD symptoms [31,32]. In this work, we used the data from the following measurements:

- Unified Parkinson’s Disease Rating Scale (UPDRS III): covers the motor evaluation of disability;
- Montreal Cognitive Assessment (MoCA): assesses different types of cognitive abilities;
- University of Pennsylvania Identification Test (UPSIT): determines an individual’s olfactory ability;
- State-Trait Anxiety Inventory (STAI); diagnoses anxiety and distinguishes it from depressive syndromes;
- Geriatric Depression Scale (short form, GDS): identifies depression symptoms.

### 2.2. Data Dimension Reduction: Principal Component Analysis (PCA) and Linear Discriminant Analysis (LDA) Techniques

Feature reduction is a process of linear or nonlinear transformation of the raw space into a small subset of features [33,34,35]. This transformation diminishes the computational complications of learning algorithms. Both PCA and LDA are linear feature reduction techniques that are commonly used for dimensionality reduction. PCA is an unsupervised dimensionality reduction process [34]. It projects the data to a newly generated system of coordinates in such way that the highest variance by any projection of the data is on the primary dimension, the second greatest variance on the secondary dimension and so on. Contrary to PCA, LDA is a supervised dimensionality reduction method that projects a dataset into a shorter subspace while retaining the class discriminatory information [35]. It calculates the linear discriminants that denote the directions that maximize the separation across several classes.

The steps involved in PCA and LDA dimensionality reduction techniques are represented by Algorithms 1 and 2, respectively [33,34,35].
**Algorithm 1:** PCA steps**1**: Ignore the dataset (consists of d-dimensional sample) class labels.**2**: Calculate the d-dimensional mean vectors: the mean for every dimension of the whole dataset. The mean vector is computed by the following equation:$m=1/n\sum_{k=1}^{n}x_{k}\;\;\;\;\;\;\;\;\;\;\;\;\;\;\;\;\;\;\;\;\;\;$ (1)**3**: Calculate the scatter matrix or the covariance matrix of the dataset. The mean vector is computed by the following equation:$S=\sum_{k=1}^{n}(x_{k}-m)\;{\left(x_{k}-m\right)}^{T}\;\;\;\;\;\;\;\;\;\;\;\;\;\;\;\;\;\;$ (2)**4**: Calculate the eigenvectors and corresponding eigenvalues of the covariance matrix.**5**: Sort the eigenvalues by decreasing eigenvalues and pick k eigenvectors with the largest eigenvalues to form a *d* × *k* dimensional matrix *W* of eigenvectors.**6**: Use the *W* eigenvector matrix to transform the sample (original matrix) into the new subspace via the equation: $y=W^{T}x\;\;\;\;\;\;\;\;\;\;\;\;\;\;\;\;\;\;\;\;\;\;\;\;\;$ (3)where *x* is a d × 1-dimensional vector representing one sample and *y* is the transformed *k* × 1-dimensional sample in the new subspace.

In this work, we used PCA and LDA techniques because of the relatively high number of features in the PPMI dataset.
**Algorithm 2:** LDA steps**1**: Compute the d-dimensional mean vectors of the dataset classes:$m_{i}=1/n_{i}\sum_{x\in D_{i}}^{n}x_{k}\;\;\;\;\;\;\;\;\;\;\;\;\;\;\;\;\;\;\;\;$ (4)**2**: Compute the scatter matricesbetween-class and within-class scatter matrix.The within-class scatter matrix $S_{W}$ is computed by the following equation:$S_{w}=\sum_{i=1}^{c}S_{i}\;\;\;\;\;\;\;\;\;\;\;\;\;\;\;\;\;\;\;\;$ (5)$where\;\;\;S_{i}=\sum_{x\in D_{i}}^{n}\left(x-m_{i}\right){\left(x-m_{i}\right)}^{T}\;\;\;\;\;\;\;\;\;\;\;\;\;\;\;$ (6)The between-class scatter matrix $S_{B}$ is computed by the following equation:$S_{B}=\sum_{i=1}^{c}\;N_{i}\left(m_{i}-m\right){\left(m_{i}-m\right)}^{T}\;\;\;\;\;\;\;\;\;\;\;\;\;\;\;\;$ (7)where **m** is the overall mean, and $m_{i}$ and $N_{i}$ are the sample mean and the size of the respective classes.**3**: Compute the eigenvectors and associated eigenvalues for the scatter matrices.**4**: Sort the eigenvectors by decreasing eigenvalues and select k eigenvectors with the highest eigenvalues to form a d x k dimensional matrix W.**5**: Use the W eigenvector matrix to transform the original matrix onto the new subspace via the equation:y=X W                      (8)where *X* is an n × d-dimensional matrix representing the n samples, and *Y* is the transformed *n* × k-dimensional sample in the new subspace.

### 2.3. Clustering Algorithms: K-means, DBSCAN and Hierarchical Clustering

Clustering is an unsupervised learning technique that is effectively applied in various fields such as data mining and image analysis [36,37]. It is used for partitioning an unlabeled set into clusters based on similarities in the same cluster and dissimilarities between different clusters; data in the same cluster are more similar to each other than in different clusters [36]. Typically, similarity of data is compared using a distance measure [37]. Various types of clustering algorithms are proposed to suit different requirements. In this work, three clustering algorithms, namely K-means, DBSCAN and Hierarchical Clustering, were used to identify PD patients within the SWEED set.

#### 2.3.1. K-means Algorithm

K-means algorithm, also known as the K-nearest-neighbor algorithm, is a clustering approach applied to cluster data into k partitions based on the distance between different input data points. Every cluster is defined by its centroid [36]. The centroid is the point whose coordinates are calculated by computing the average of each of the coordinates of the sample points affected to the clusters. It is computed as follows:

(9) $$c=1/N_{c}\sum_{j=1}^{N_{c}}\;x_{j}\;\;$$

where $N_{c}\;$is the vector’s number in the subset.

The centroid data point distances are computed by the Euclidean distance, cosine dissimilarity or by other distance functions.

The steps involved in the K-means method are represented by Algorithm 3.
**Algorithm 3:** K-means steps**1**: Select the required number of clusters, k.**2**: Select k starting points to be used as initial estimates of the cluster centroids.**3**: Attribute each point in the database to the cluster whose centroid is the nearest.**4**: Recalculate the new k centroids.**5**: Repeat steps 3 and 4 until no data point changes its cluster assignment or until the centroids no longer move (until the clusters stop changing).

#### 2.3.2. Density-Based Spatial (DBSCAN) Algorithm 

The Density-Based Spatial (DBSCAN) algorithm is a clustering algorithm that does not depend on a predefined number of clusters. Data are divided into areas of high density (clusters) separated from each other by areas of low density (noise) [36]. An area is dense if it includes at least N patterns at a distance R for a given N and R. This approach considers two input variables: ϵ and Nmin. The parameter ϵ defines a neighborhood of the input data xi. The minimum point parameter Nmin determines a center object, a point with a neighborhood composed of more elements than this parameter. The steps involved in the DBSCAN approach are illustrated in Algorithm 4.
**Algorithm 4:** DBSCAN steps**1**: The algorithm begins with a random sample in which neighborhood information is taken from the ϵ parameter.**2**: If this sample contains Nmin within ϵ neighborhood, cluster formation starts. Otherwise, the pattern is marked as noise or may later be found in the ϵ neighborhood of a different pattern and, hence, can be incorporated into the cluster. If a sample is found to be a core point, then the samples within the ϵ neighborhood are also part of the cluster. So, all the samples found within ϵ neighborhood are added, along with their own ϵ neighborhood, if they are also core points.**3**: The process (step 2) restarts with a new point, which can be a part of a new cluster or labeled as noise, skipping every sample already assigned to a cluster by the preceding iterations. After DBSCAN completes the data processing, each sample is assigned to a particular cluster or it is an outlier.

#### 2.3.3. Hierarchical Clustering

There are two main types of Hierarchical Clustering, namely agglomerative (also known as AGNES: Agglomerative Nesting) and divisive (known also as DIANA: Divisive Analysis) [37]. The agglomerative type uses a bottom-up approach to form clusters. It starts with single-element clusters, and then distances between these clusters are calculated and the clusters that are closest to each other are merged. The same process repeats until one single cluster is obtained. Divisive Hierarchical Clustering is the opposite of agglomerative Hierarchical Clustering. It is a top-down approach that starts from one single cluster and then divides the farthest cluster into separate clusters; at each step of the iteration, the most heterogeneous cluster is divided into two. The process is continued until all elements are in their own cluster.

The Hierarchical Clustering result is often plotted as a dendrogram. Nodes in the dendrogram represent clusters, and the length of an edge between a cluster and its split is proportional to the dissimilarity between the split clusters. In fact, the y coordinate shows the distance between the objects or clusters.

## 3. Results

### 3.1. Data Dimension Reduction: Feature Extraction Based on PCA and LDA Techniques

**Figure 3:**
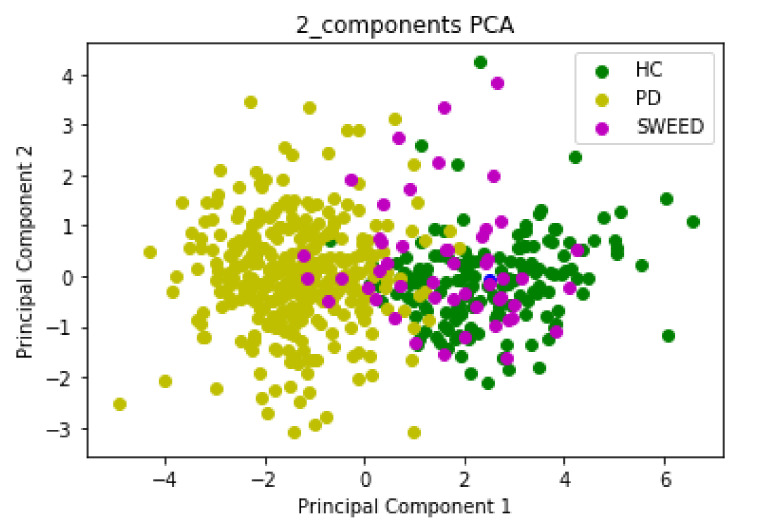
The new feature subspace constructed using PCA with class labels.

**Figure 4:**
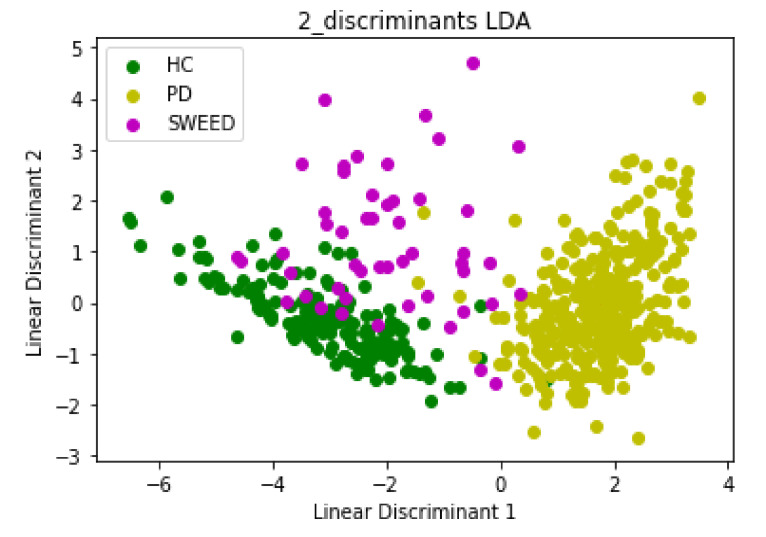
The new feature subspace constructed using LDA with class labels.

Input data for PCA and LDA algorithms consisted of 548 rows and 9 columns; we started with a 9D dataset that we reduced to a 2D dataset by dropping seven dimensions. The first Principal Component (PC1) and the second Principal Component (PC2) had eigenvalues > 1 (PC1 = 4.6 and PC2 = 1.1), which were sufficient to describe the data and reduce the complexity of data analysis. The scatter plot in Figure 3 and Figure 4 represents the PCA and LDA projections, respectively, of PD, HC and SWEDD groups.

### 3.2. Clustering Algorithm: K-means, DBSCAN and Hierarchical Clustering

**Figure 5:**
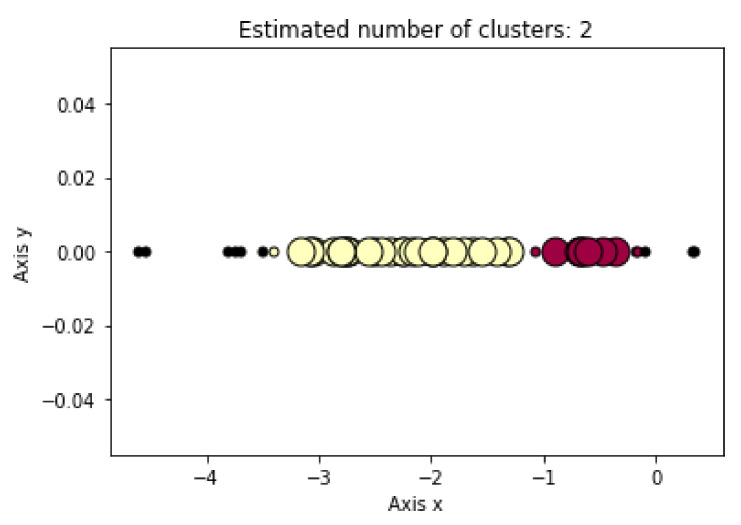
Distribution of predicted DBSCAN clustering result using LD1 as input.

**Figure 6:**
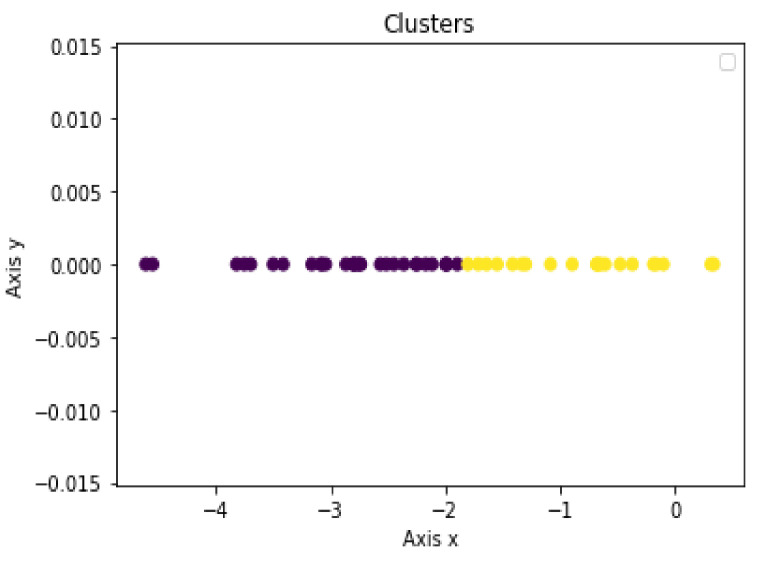
Distribution of predicted K-means clustering result using LD1 as input.

**Figure 7:**
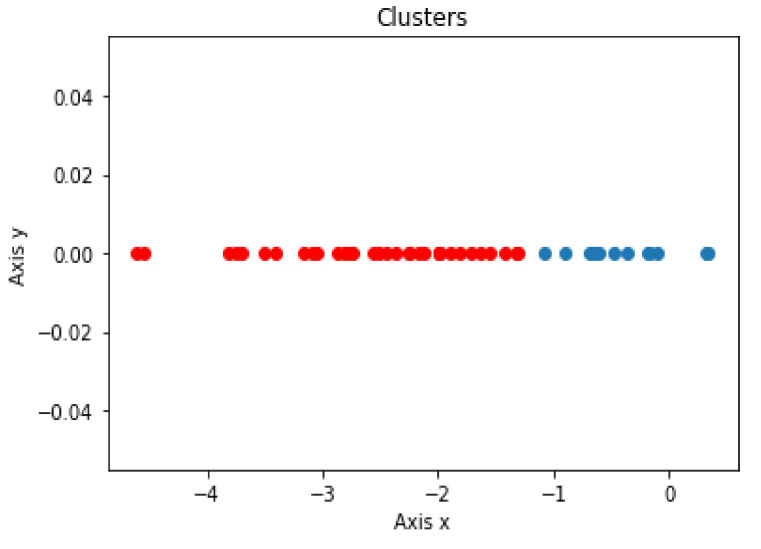
Distribution of predicted Hierarchical Clustering result using LD1 as input.

The LD1 new feature subspace of the SWEDD group was used as the input for the unsupervised learning analysis (K-means, DBSCAN and Hierarchical Clustering). These algorithms were employed to automatically generate two clusters, PD and HC. Figure 5, Figure 6 and Figure 7 present the K-means, DBSCAN and Hierarchical Clustering algorithms plots, respectively. As the output algorithms were one-dimensional data, we plotted them in a horizontal axis (one-dimensional graph) with the *y*-axis set to zero. Each clustering algorithm was designated two distinct distributions with different colors representing two clusters that corresponded to PD and HC groups.

In order to identify the corresponding group of each cluster, we calculated the Euclidean distance between each point and the centroid of each group (PD and HC). Then, we assigned each point to the closest group.

Using the DBSCAN algorithm, 11, 31 and 9 subjects were identified as HC subjects, PD patients and noise, respectively.

Using the K-means algorithm, 21 and 30 subjects were identified as HC subjects and PD patients, respectively.

Using the Hierarchical Clustering algorithm, 15 and 36 subjects were identified as HC subjects and PD patients, respectively.

**Figure 8:**
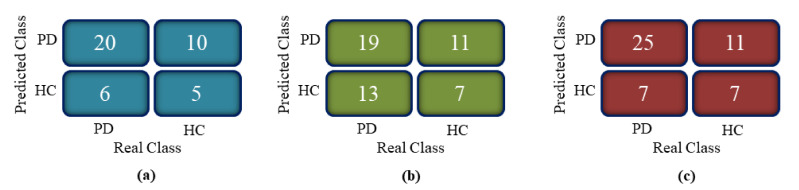
Confusion matrix of clustering algorithms: (**a**) DBSCAN, (**b**) K-means and (**c**) Hierarchical Clustering.

In fact, after two years of follow-up with 51 subjects from SWEDD, 32 patients demonstrated a reduced dopamine uptake on DaTscan SPECT, whereas 18 subjects continued to have normal dopaminergic imaging (HC) and one subject remained SWEDD. Figure 8 shows the confusion matrices of the clustering models. 

From the confusion matrices, performance measures in terms of accuracy, specificity, sensitivity and F1 score were computed. Table 3 shows the performance of each clustering algorithm.

## 4. Discussion

The emergence of Machine Learning (ML) algorithms to identify hidden patterns in complex and multidimensional data has offered unparalleled opportunities for numerous researchers to assist in Parkinson’s Disease (PD) diagnosis. These algorithms are able to distinguish between Healthy Control (HC) subjects and PD patients within Scan Without Evidence of Dopaminergic Deficit (SWEDD) groups. However, there is an overlap among the three groups in both clinical and Single-Photon Emission Computed Tomography (SPECT) imaging features. The amount of overlap of these groups in clinical and imaging features determines the difficulty of the classification problem. In this research work, an automatic diagnosis system based on clustering models that involved nine variables was developed to separate PD patients from HC subjects within an SWEDD group. We considered SBR values of the four striatal regions as imaging features and UPDRS III, MoCA, UPSIT, STAI and GDS SHORT as clinical features. These variables were reduced by PCA and LDA algorithms that sought the directions of greatest variation in the dataset. They eliminated and removed recursive and redundant data and retained important information with very minimal loss: out of nine features, two were selected to be the significant contributors. The comparison of these two popular subspace projection methods showed the superiority of the LDA algorithm. These results are explained by the fact that PCA is less optimal for scattering interclass and removed only the information concerning the linear structure in data. The LDA method overcame PCA’s shortcomings and removed the nonlinear structure. It maximized the value of between-class scatter and the value of within-class scatter. In addition, the first Linear Discriminant (LD1) and PC1 separated the classes nicely. Nevertheless, the second Linear Discriminant (LD2) and PC2 did not contribute much additional valuable information. As LD1 was the most significant axis, it was retained and used as an input for the three popular clustering algorithms (DBSCAN, K-means and HC). These algorithms classified SWEDD subjects into two subtypes, PD and HC. The performance of each algorithm was evaluated by comparing the class assigned to each subject (clustering output) with the standard of truth given by the diagnosis at the last available follow-up. In fact, SWEDD subjects were clinically followed up and evaluated, and the initial SPECT neuroimaging data were labeled. After the follow-up period, there was variability within the SWEDD group; about 70% (35 subjects) experienced a decline in SBR, which confirmed the PD disease, while the other 30% (15 subjects) demonstrated a slight rise in SBR from baseline, which confirmed that these subjects were normal. The slight rise in SBR in normal subjects is explained by the fact that these subjects were on PD medication. The comparison of the standard of truth and the clustering output showed that the truth does not totally coincide with predicted PD. This difference between the truth and the prediction reflects the error rate of clustering algorithms. The three clustering algorithms produced close results, and the highest performance was obtained by the Hierarchical Clustering algorithm, with an accuracy of (64%), sensitivity of (78.13%), specificity of (38.89%) and F1 score of (73.53%). Indeed, the proposed method revealed a clear separation between PD and HC within the SWEDD group based on clinical and imaging features.

The related research studies [18,19,20,33,34,35,36] used data with a different number of subjects. Consequently, results are not directly comparable. However, as an indication, the studies of Nicolas Nicastro et al. [33] achieved less accuracy (58.4%) and sensitivity (45%) in identifying PD compared to our model (accuracy of 64% and sensitivity of 78.13%). The approaches proposed in the research studies [18,19,20,34,35,36] achieved good accuracies, but their algorithms were used for classification of PD and HC groups and also implemented computationally intensive models. However, our approach distinguishes PD from HC within an SWEDD group by implementing a computationally simple model. In addition, it used a large dataset with a high number of heterogeneous features. These features were optimized and selected using feature reduction techniques to retain important data, eliminate recursive and redundant information and speed up the execution time, which makes the model more robust. 

Despite the promising results of the proposed method, which is fast, requires little user intervention and can be easily extended to a clinical setting, it has several sources of misclassification which should be specifically considered here: First, we limited the clinical analysis to just five features, and imaging features analysis to four features, which did not cover all the clinical features and the entire brain. Likewise, SPECT is the practical option for assessing PD patients. However, the brain can be assessed not only using SPECT, but also by other imaging modalities, including MRI and PET. Hence, multimodel brain examination can permit the integration of supplementary information from various modalities to enhance PD patients’ differentiation from HC subjects within an SWEDD group. Moreover, despite the relatively large number of samples in the dataset used in this study, we speculate that the number of subjects in the SWEDD group was too limited to capture the full variability in clinical and imaging features. In fact, to date, ground truth and the follow-up of SWEDD groups is difficult to achieve. Additionally, imbalanced data from the two classes within the SWEDD group are associated with lower classification accuracy in the minority class.

## 5. Conclusions

The focus of this study was to separate PD patients from HC subjects within an SWEDD group using clinical assessment and DaTSCAN SPECT imaging features. The features were first minimized by two dimensionality reduction techniques, PCA and LDA, to find a lower-dimensional subspace; 2D nine features were adjusted to two 1D features, but it still retained the information of the large set. Indeed, the strongly correlated features were obtained with the LDA algorithm. Thus, the LDA-reduced set was analyzed through the clustering models DBSCAN, K-means and Hierarchical Clustering. Each clustering algorithm produced two subsets within the SWEDD group (PD and HC). The different clustering performance metrics were evaluated by comparing the clustering algorithms outcomes with the known ground truth. In fact, after the follow-up, 70% (35) of the SWEDD subjects versus only 30% (15) demonstrated a dopamine decline from baseline (had lower SBR scores). Hierarchical Clustering exceeded DBSCAN and K-means algorithms and achieved an accuracy of 64%, a sensitivity of 78.13%, a specificity of 38.89% and a F score of 73.53%. These promising results show that the separation between early PD patients and HC subjects within the SWEDD group based on clinical and SPECT imaging features in the cohort of 548 subjects can be adequately addressed by an automatic system using ML. In future research works, different feature reduction and clustering methods, as well as other types of data, such as motor test data and motion sensors for movement detection, will be examined to improve the performance metrics. We also aim to explore deep learning models for early PD identification as they show promising results in classification and detection issues.

## Figures and Tables

**Table 1:** Summary of existing classification approaches for PD diagnosis.

Authors	Objectives	Sample Size	Features	Methods	Accuracy
Diego et al. (2018) [21]	Classify PD patients and HC subjects	388 subjects obtained from PPMI database	Morphological features extracted from DaTSCAN images with biomedical tests	SVM classifier with LOO-CV method	96%
Nicolas Nicastro et al. (2019) [24]	Distinguish PD patients from other parkinsonian syndromes and HC subjects	578 subjects (local database)	Semi-quantitative 123-FP-CIT SPECT uptake values	SVM with five-fold CV method	58.4%
Yang et al. (2020) [22]	Classify PD patients and HC subjects	101 subjects taken from PPMI dataset	Multimodel neuroimaging features composed of MRI and DTI with clinical evaluation	SVM, Random Forests, K-nearest Neighbors, Artificial Neural Network and Logistic Regression with ten-fold CV method	96.88%
Dotinga et al. (2021) [23]	Distinguish PD patients from non-PD subjects	210 subjects	SBR values computed from I-123 FP-CIT SPECT, age and gender	SVM with ten-fold CV method	95%
Lavanya Madhuri Bollipo et al. (2021) [25]	Classify early PD patients and HC subjects	600 subjects obtained from PPMI dataset	Clinical scores, SBRs values and demographic information	Incremental SVM with LOO-CV method	98.3%
Lavanya Madhuri Bollipo et al. (2021) [26]	Distinguish early PD patients from HC subjects	634 subjects taken from PPMI dataset	Motor, cognitive symptom scores and SBR values computed from DaTSCAN	SVR	96.73%
Diego Castillo-Barnes et al. (2021) [27]	Distinguish PD patients from HC subjects	386 samples selected from PPMI database	Morphological features computed from 123I-FP-CIT SPECT	SVM, Naive Bayesian and MLP with ten-fold CV method	97.04%

**Table 2:** Means of clinical and imaging features of subjects.

	HC	SWEDD	PD
Number	156	51	341
SBR (Best Putamen)	2.26	2.17	0.97
SBR (Worst Putamen)	2.04	1.89	0.66
SBR (Best Caudate)	3.08	2.94	2.16
SBR (Worst Caudate)	2.85	2.72	1.79
UPDRS III	1.20	14	20.61
MoCA	28.20	27.16	26.59
UPSIT	34.03	31.37	22.12
STAI	−0.24	0.04	0.09
GDS	5.15	5.71	5.26

**Table 3:** DBSCAN, K-means and Hierarchical Clustering performance.

Measure	DBSCAN	K-means	Hierarchical Clusternig
Accuracy %	60.98	61.29	64.00
Sensitivity %	76.92	59.38	78.13
Specitivity %	33.33	38.89	38.89
F1 score %	71.43	61.29	73.53

## Data Availability

Not applicable.
